# Visual consciousness dynamics in adults with and without autism

**DOI:** 10.1038/s41598-022-08108-0

**Published:** 2022-03-14

**Authors:** Jan Skerswetat, Peter J. Bex, Simon Baron-Cohen

**Affiliations:** 1 Anglia Vision Research, Department of Vision and Hearing Sciences, Anglia Ruskin University, Cambridge, UK; 2 Department of Psychology, Northeastern University, Boston, USA; 3 Autism Research Centre, Department of Psychiatry, University of Cambridge, Cambridge, UK

**Keywords:** Pattern vision, Human behaviour, Autism spectrum disorders, Learning algorithms

## Abstract

Sensory differences between autism and neuro-typical populations are well-documented and have often been explained by either weak-central-coherence or excitation/inhibition-imbalance cortical theories. We tested these theories with perceptual multi-stability paradigms in which dissimilar images presented to each eye generate dynamic cyclopean percepts based on ongoing cortical grouping and suppression processes. We studied perceptual multi-stability with Interocular Grouping (IOG), which requires the simultaneous integration and suppression of image fragments from both eyes, and Conventional Binocular Rivalry (CBR), which only requires global suppression of either eye, in 17 autistic adults and 18 neurotypical participants. We used a Hidden-Markov-Model as tool to analyze the multistable dynamics of these processes. Overall, the dynamics of multi-stable perception were slower (i.e. there were longer durations and fewer transitions among perceptual states) in the autistic group compared to the neurotypical group for both IOG and CBR. The weighted Markovian transition distributions revealed key differences between both groups and paradigms. The results indicate overall lower levels of suppression and decreased levels of grouping in autistic than neurotypical participants, consistent with elements of excitation/inhibition imbalance and weak-central-coherence theories.

## Introduction

Autism spectrum condition (henceforth autism) refers to a diagnosis where the person faces social and communication challenges, alongside restricted interests and repetitive behaviors, and difficulties adjusting to unexpected change (American Psychiatric Association, 2018). There are well-known sensory differences between autistic and typical people, including in visual perception^1–5^, hence sensory sensitivities have recently been added to hallmark features of autism diagnosis^6^.

Although there is limited evidence for any consistent difference between neurotypical and autistic people in sensory thresholds for detecting isolated features, there is compelling evidence for differences in combining features across space or time^1^. For example, autistic individuals are less sensitive to global form^7^ or motion^8^ and are biased towards the local features in complex figures^9,10^. These observations led to the Weak-Central-Coherence theory^11^ of autism, which has been tested using various spatial integration and perceptual grouping methods, with mixed results^12–14^.

These perceptual differences are thought to be a consequence of an excitation/inhibition(E/I) imbalance across the cortex^15–17^. (See also in-depth review on the literature concerning reduced gamma-aminobutyric acid (GABA, an inhibitory neurotransmitter) and its role in the context of autism^15^). It is however noteworthy that these differences may be brain region- and/or age-specific as a magnetic resonance spectroscopy study in children with autism only found differences in motor and auditory, but not visual brain areas^18^. Similarly, another magnetic resonance study in children with autism investigated GABA concentrations during a visual search task in the right frontal eye fields, right temporal–parietal junction, and bilateral visual cortex. The researchers did not find GABA difference between groups, but increased GABA levels in the visual cortex that were correlated with more efficient search in the autism group^19^.

**Figure 1:**
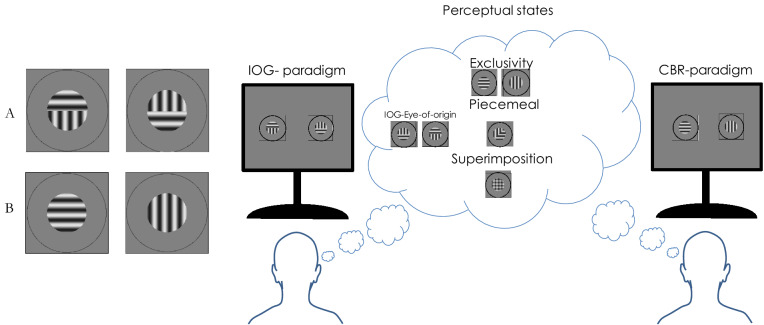
Example stimuli used to initiate IOG (**A**) and CBR (**B**) in the current study and their respective perceptual experiences. The normally sighted reader may be able to experience both rivalry and grouping. Start by looking at the gap between the gratings in A or B in each row at reading distance. Position a fingertip halfway between your eyes and the gratings, so that each eye views the fingertip centrally for one of the two gratings. Now fixate the fingertip with both eyes open and three, overlapping gratings should be perceived. The central patch may compete and group perceptually. This figure was composed using Microsoft PowerPoint Software and the images of grating stimuli were generated using Matlab 2021 Software.

Cortical excitation and inhibition have been investigated behaviourally with interocular visual suppression provoked by conventional binocular rivalry (CBR), a paradigm in which dissimilar images are presented to each eye simultaneously. During CBR, observers experience ongoing alternations of perception among four principal perceptual states: the exclusive appearance of the image presented to the left eye, the exclusive appearance of the image presented to the right eye, a piecemeal mixture of both images, or a superimposition of both images (see Fig. 1B).

Within the context of perception during CBR, the two classes of mixed states, namely piecemeal^20^ and superimposition, have been thought to be processed by different neural populations. The underlying neural correlate of superimposition has been suggested to be a product of binocular combination neurons^21–25^. A neuroimaging study^24^ used fusible, semi-fusible i.e., fusible stimuli with embedded rivalrous elements, and rivalrous stimuli to measure Blood-Oxygen-Level-Dependent (BOLD) changes along the visual pathway areas in V1-V4 and beyond. Two key findings were that the effect decreased along the visual pathway, and that adding a rivalrous element to an otherwise fusible stimulus, increased BOLD signal only in V1 and V2 but not in V3 or V4, implying that rivalry predominantly affects early visual cortex where many neurons are monocular. Katyal et al.^25^ measured Visually Evoked Potentials (VEPs) during CBR with monocularly tagged stimuli and reported intermodulation responses, i.e. combinations of the two input frequencies, which can only be evoked by binocular neurons. The researchers found that both the two single and the intermodulation frequencies were predominately observed at electrodes close to the primary visual cortex, and found peak amplitudes of intermodulations during phases of mixed perception, suggesting that binocular neurons cause this perceptual state. Based on their finding that rivalry mixed and exclusivity is related to both eccentricity, spatial frequency and stimulus size, Blake and collaborators^20^ suggested that piecemeal experiences are the result of multiple, monocular zones of rivaling stimuli that are seen simultaneously. However, neuro-imaging studies found activation of regions at the early visual cortex, but also beyond; in particular in parietal and frontal cortex^26,27^. Psychophysical studies concerning ocular deprivation and its effect on CBR^23,28^ and CBR in luminance and contrast modulated stimuli^29^ demonstrated difference between piecemeal and superimposed perception. It is not clear whether piecemeal and superimposition is a result of simultaneously conscious awareness of the different monocular sites, or a result of a higher-level representation beyond the monocular level. However, in the light of the psychophysical differences between piecemeal and superimposition mentioned above, it is reasonable to assume that the processing mechanism of piecemeal differs from that of superimposition, as well as from exclusive perception.

Several studies have investigated CBR in autism^30–36^, including mixed perception, but have not classified piecemeal or superimposition components. The first study concerning CBR and autism did not find a significant difference between autistic and control groups^30^. However, that study employed a large stimulus size, which is known to be biased toward mixed perception^20^. Later studies used smaller apertures and found longer durations of mixed perceptions (and fewer perceptual alternations) in adults with autism^31–33^ and in neuro-typical adults with an elevated autistic symptomology^36^. Several studies have found that stereoacuity is reduced in autism compared to controls^37,38^ and people with autism are more likely to have binocular vision disruptions due to strabismus and amblyopia^2,38–42^.

It is possible that binocular sensory differences between participants with and without autism may contribute to differences in mixed perception during CBR. Our first aim was therefore to investigate whether perception of piecemeal and superimposition differs both within autism and compared to controls. It is noteworthy that one study found no significant differences between children with and without autism^34^, which the authors speculated may be either due to a weaker excitation/inhibition imbalance in children with autism, or attenuated top-down control in autistic children. In adults, Robertson et al.^33^ found that glutamate levels (an excitatory neurotransmitter) in early visual cortex correlate strongly with proportions of perceptual suppression in CBR in both autistic and control cohorts. In contrast, GABA levels only correlated with suppression perception in controls. A recent electrophysiological study confirmed that autistic people experience fewer perceptual alternations in CBR and found that alternations were correlated with reduced neuronal alternation for autism compared to controls^35^.

To explore excitation/inhibition and spatial grouping in greater detail, we used a paradigm called interocular grouping (IOG)^43,44^ that simultaneously provokes suppression and grouping of two partially dissimilar binocular images to investigate how grouping and suppression interact in autistic compared to neurotypical adults. In IOG, each eye’s stimulus is composed of two bipartite images (see Fig. 1A). IOG can elicit the same perceptual experiences as CBR, however note that exclusive percepts of one pattern (here a grating), requires inhibition of part of the image from each eye, indicating a late stage grouping and suppression mechanism. IOG therefore provides a powerful tool to investigate the dynamics and processing stages of excitation and inhibition. Intuitively, IOG was thought to be a product of a binocular mechanism rather than monocular (i.e. eye-of-origin) mechanism that interocularly combines portions from each image to create a coherent cyclopean percept^44^. To examine the cortical locus of this suggested binocular mechanism, Stuit et al.^45^ examined grouping of inverted and upright face stimuli, which are thought to be processed at mid-level and high-level processing stages, respectively. They found no significant difference between IOG for inverted and upright faces^45^ or grouped face and grating stimuli. These observations suggest that IOG is not moderated by feedback from higher-level face-processing areas but instead by lateral connection between mid-level processing stages. Said and Heeger^46^ argued that opponency neurons that receive excitatory input from one and inhibitory input from the other eye are required to explain CBR dynamics. If true, this may extend to IOG as well^47^. We therefore hypothesized that IOG perception would differ between neurotypical and autistic participants because of the imbalance of excitation/inhibition across the cortex^15–17^.

The effect of physical changes of the stimuli on the perception for both IOG^44,47,48^ as well as CBR (e.g. Breese, 1899, 1909;^51^ have been studied intensively. The relation between physical properties of either one or both rival stimuli and their influence on perception during CBR has been lawfully described by Levelt^51^, and was subsequently updated by Brascamp, Klink and Levelt^52^. Recent studies showed that whether or not IOG perception obeys Levelt’s laws also depends on stimulus visibility and hence obeying Levelt’s first three laws when changing unilateral chromatic stimuli’s colour saturation^48^ and stimulus type^47^. No study investigated however whether these laws could be replicated for autistic participants during either CBR or IOG. Therefore, we aimed to investigate whether physical changes and their relation to perception was affected in people with autism. An important framework that has been suggested to mediate both CBR and IOG is mutual inhibition, i.e. the alternation of perception is a product of an ongoing increase and decrease of inhibition/excitation of eye-of-origin neurons. We therefore varied the stimulus contrast both unilaterally-and bilaterally to test whether the suggested E/I imbalance in autism would show differences in outcomes. We also manipulated unilateral and bilateral contrasts because superimposition during CBR increases when the contrasts of the rival gratings are lowered simultaneously^21,22^ and there is also a decrease of the alternation between exclusive percepts^52^.

IOG and CBR have been traditionally reported via four parameters, namely relative proportions and their respective mean durations for each perceptual states, the number of perceptual alternations between those states, in particular the changes between exclusive states, and the distribution of the exclusively visible events in relation to a gamma function^44,49–51^. However, those measures do not capture alternations, means, and relative proportions on a specific perceptual state-to-state basis but rather are typically averages across all or exclusive-to-exclusive combinations, nor do they capture the likelihoods of those individuals changes to occur. As discussed above, the different perceptual states very likely underly distinct neural correlates, therefore a method that reveals such difference with greater resolution and computational provides greater insights in the dynamics of rivalry.

**Figure 2:**
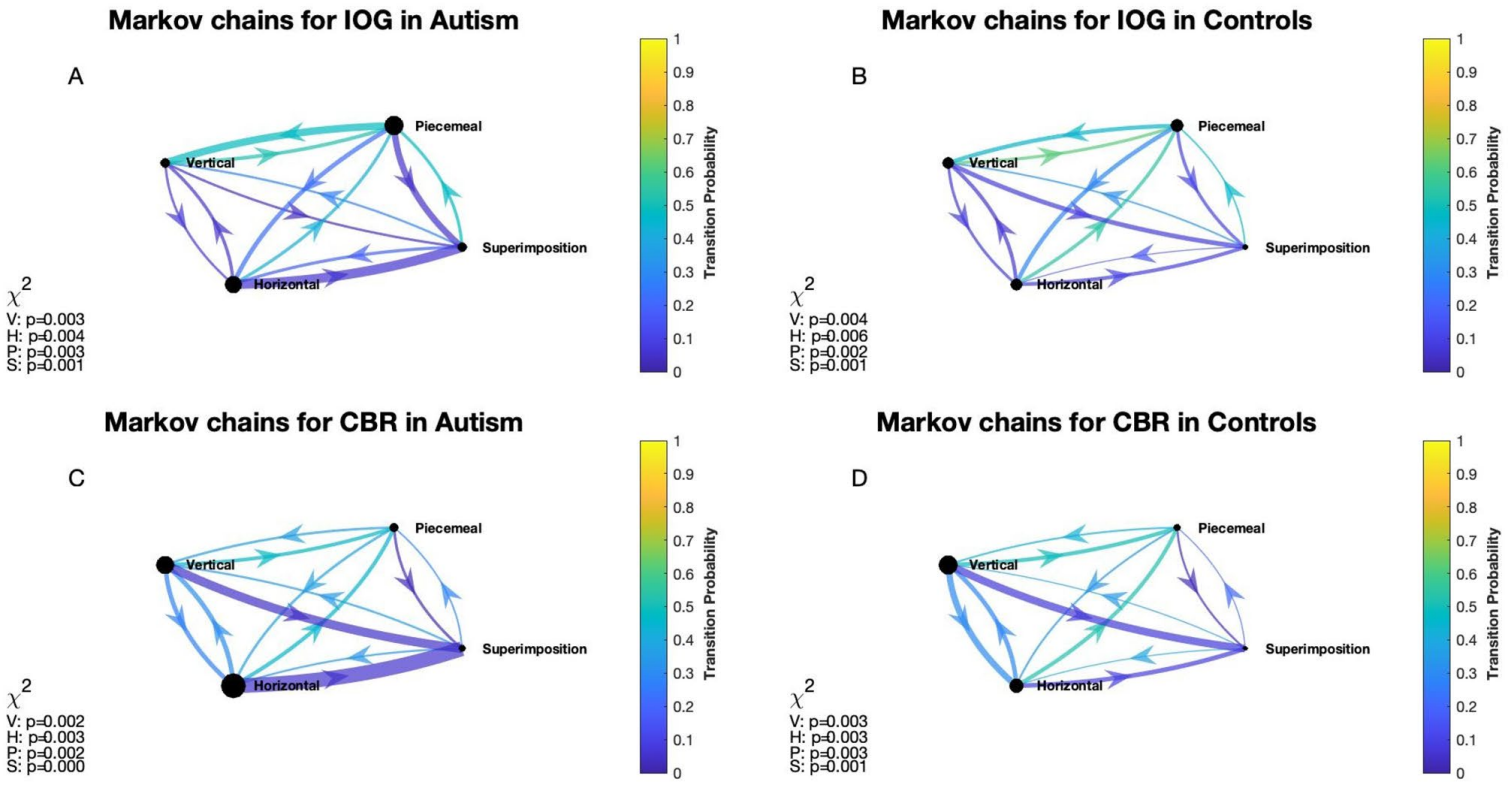
Markov chain models of grouping and rivalry dynamics for autism and neurotypical observers. Each figure shows the Markov chain for averages across trials, participants, and contrast conditions for autism (left column) and controls (right column) for IOG (top row) and CBR (bottom row) paradigms. The transition probability is indicated via line colour and colour bar, the transitions that occurred are indicated via arches with arrows indicating the direction of transition, the diameters of the nodes represent the mean durations for the four perceptual states and the thickness of each arch represent the mean durations for each individual transition before (actual) alternation to the other perceptual states (posterior). The chi-square values on the left-hand side of each chain are variance test results and show that the transition probabilities for each state were significantly different from a normally distributed probability, here 33%.

Hidden-Markov-Models (HMM) generate transition probabilities on a state-to-state basis and are typically depicted as chains showing states, each transition path that occurred, and their likelihood to occur. We use a modified version of HMM chains that address these issues by depicting each perceptual state, the observed transitions and their likelihoods, based on a Markovian model, and the direction of a transition (e.g. left exclusive to piecemeal versus piecemeal to left exclusive transition). Additionally, we implemented weights of transitions, calculated as the mean duration prior to a transition and depicted weights via arrow width (i.e. the thicker the arrow the longer the prior mean duration) as well as the mean duration of each state. This degree of computational detail distinguishes the HMM chains from other CBR analysis methods. As seen in Fig. 2, the colour scheme indicates biases of likelihood of transition in certain directions, further tested statistically by the chi-square values in the figure. Next to the greater resolution of data analysis in comparison to conventional analysis, the HMM provides an alternative computational approach to predict of transitions during CBR. Various computational models of rivalry have been suggested that are meant to explain the nature of transitions. We therefore use Hidden Markov models as a computational tool to model multistable perception for both IOG and CBR and to compare autistic and neurotypical observers.

In summary, the current study aims to address four questions:

First, how does the perception of IOG differ between people with autism and neurotypical controls? According to the E/I and weak central coherence theories, we hypothesize that the autism group will experience fewer IOG percepts whose mean duration would be shorter than that of the control group.

Second, what is the differential role of piecemeal and mixed perception in people with autism.

compared to the control group? For the same reason described for question one, we expect piecemeal duration to be longer in autism than in the control group. As we control for normal binocular vision, we remain agnostic as to whether superimposition would or would not be significantly disrupted in autism compared to controls. We hypothesize that there will be a reduced proportion, numbers, and mean duration of superimposition events in the ASC group compared to the control group.

Third, what is the difference in probability of perceptual connections using a Markovian approach? We would expect the probabilities of perception to vary along the lines of the difference described in questions one and two. Hence, the chain connections (e.g. horizontal to piecemeal) may be the same for each group, but their probability may differ. Specifically, we expected the probabilities to be highest in perceptual connection with piecemeal in the autism group and further to be higher than those of the control group.

Fourth, what are the effects of contrast in IOG and CBR perception for people with autism compared to the control group? The effects of contrast on CBR can be estimated by applying Levelt’s laws^51,52^ and have been previously extended to IOG paradigm^47,48^. It is unclear whether different unilateral and bilateral stimulus energy levels may cause differential effects in autism people’s perception compared to that of controls. If E/I imbalance theory causes perceptual difference in autism, then we hypothesize that it would be reflected in difference in the patterns of perceptual reports. Specifically, the laws may hold for autism but the degree to which contrast affects IOG changes in autism may be different from that of the control group. A previous study in a general cohort found that exclusive IOG percepts occur less often, with shorter mean duration, and as a lower overall proportion of perception than do exclusive percepts during CBR^44^. As we expect that IOG may be disrupted in autism, we expected the trend to be the same in both groups but with a great magnitude of difference in the autism group.

## Methods

### Participants

Initially forty-two adult participants (18 years and older) were recruited via the Cambridge Autism Research Database (CARD), online advertisements, and poster advertisements in the facilities of the Anglia Ruskin University, Cambridge, UK (Table 1). The general exclusion criteria were a diagnosis with attention deficit syndrome or epilepsy. To be included in the autism group, participants had to be diagnosed by a clinician, i.e. indicated by self-report of participant, and additionally complete two questionnaires prior the experiment, namely an Autism Spectrum Quotient (AQ), and an Empathy Quotient (EQ). These questionnaire data were either gathered from the CARD database post-experiment or by manually filling out the questionnaires in the lab post-experiment. We did not get questionnaire data for one participant and therefore did not include this individual in the data analysis. One participant did not finish the task because of computer issues.

**Table 1:** Comparison of demographic and psychometric data for autism and neurotypical participants. The standard deviations (σ) as well as the ranges were indicated for age, sex, AQ and EQ with suggested threshold values of ≥ 32^53^ and ≤ 30, respectively (see also supplementary information for scatter plot). These AQ and EQ criteria were fulfilled by 16/17 and 5/17, respectively, within the autism cohort, 0/18 and 0/18 within the control group.

	Number of included and excluded participants n	Age σ (Min–Max)	Sex (M:F)	AQ σ (Min–Max)	EQ σ (Min–Max)
Autism	17 (excluded 4)	39.2 12.4 (19–61)	6:11	39.1 5.9 (22–47)	19.5 12.2 (3–47)
Control	18 (excluded 3)	28.9 6.5 (21–46)	13:5	17.7 6.5 (4–30)	47.4 9.8 (31–61)

Additionally, we included several visual/ocular exclusion criteria. Prior to the experiments, a vision screening was carried out by an optometrist to ensure regular binocular vision. It is important to control those optometric factors for the following reasons to avoid confounded raw data: first, CBR and IOG dynamics are depended on stimulus strength of the two inputs^51^; a defocus in one or both eyes may confound the data. Second, superimposed percepts during CBR have been suggested to be an indication of binocular fusion^21^, an essential feature of binocular vision. Third, autistic people show greater incidences of binocular vision issues such as strabismus and amblyopia^38,40^, which could confound the data. Fourth, stereo acuity has been shown to be reduced in autism in comparison to neuro-typical group^37^, which may affect the proportions of superimposed perception.

Hence, all participants were required to have normal or corrected-to-normal visual acuities of at least 6/6 in each eye. We also asked participants to read a Nieden chart in 1 m distance with each eye to ensure that the near acuity was not affected by potential near astigmatism or convergence/accommodation issues and all participants could read line N 12 with each eye, indicating regular near visual acuity. No participant showed an eye deviation during the cover test, to confirm the absence of strabismus. Normal binocular vision was then indicated by a random-dot-stereopsis of at least 60 arcsec when measured with the Dutch Organization for Applied Scientific Research (TNO) stereo test (Lameris Ootech, Ede, Netherlands). A Worth 4-Dot test for the distance of 1 m was carried out to test for central interocular suppression and participants had to perceive 4 lights, indicative for no central suppression. Bagolini striate lenses were used as an additional tool to test for ocular suppression and all participants saw two lines intersecting with each other, confirming the absence of central suppression. Binocular ocular motility was tested in all main gaze directions and all except one participant showed regular eye movements. That participant aligned the dichoptic stimuli prior the experiment and was therefore excluded from the study. Furthermore, four participants were excluded that had a near monocular visual acuity difference of two lines for the near and stereoacuities poorer than 60arcsec.

The current study’s screening highlighted the necessity for a thorough optometric screening prior IOG and CBR experiments as five participants had to be excluded to ensure that optometric performances differences are not the cause for CBR and IOG differences such as refraction imbalance between the eyes causing contrast differences that alter perception^52^ or disrupted binocular vision that alters perception^54^.

### Stimuli

First experiment—Interocular Grouping: horizontal and vertical sinusoidal gratings were generated with a spatial frequency of 2 c/° within a circular aperture of 2° diameter. For the left eye’s stimulus, each horizontal split-grating component was presented for four trials per condition above the vertical component and for the other four trials below the vertical component (vice versa arrangement, respectively, in the right eye’s stimulus). Both eyes’ stimuli were surrounded by a circular fusion lock with a diameter of 4° and a width of 2.6 arcmin (2 pixels) while viewed from 1 m distance through a stereoscope (see Fig. 1 A). Contrast detection thresholds in autism do not differ to neuro-typical populations^55^. Thus, we used three contrast levels of the luminance-defined gratings to test Levelt’s four laws: 0.78 vs 0.78, 0.08 vs 0.08, and 0.50 vs 0.08. Stimuli were presented on a grey background with a mean luminance of 50 cd/m^2^.

Second experiment—Conventional Binocular Rivalry: The CBR experiment started after the IOG experiment, with a longer break given. A complete horizontal grating was presented to one eye and a vertical grating to the opposite eye (Fig. 1B), counterbalancing right and left eye’s presentation for each condition. All other stimulus properties, i.e. contrasts, sizes, and spatial frequencies were the same as in the first experiment.

### Apparatus and monitor calibration

The stimuli were presented using a Mitsubishi Diamond Pro 2070SB CRT Monitor with a resolution of 1027 × 769 pixels. Dell Precision 3500 hardware and a customized MatLab program in combination with the Cambridge Research Systems Visual Stimulus Generator (ViSaGe) were used to create and present the stimuli as well as run the experiment. Gamma correction was carried out, using a Cambridge Research Systems ColorCal and software to produce lookup tables, to correct the monitor’s inherent nonlinear luminance intensities. The change of luminance after the monitor was switched on, was also measured. The results of these measurements indicated that prior to each experimental session, the monitor needed a warm-up time of 30 min to reach a consistent mean luminance level. A four-mirror stereoscope composed of optical components by OptoSigma (OptoSigma Corporation, California, USA) was used and carefully aligned prior the experiments to ensure that each eye would see only one grating.

### Procedure

Written and verbal information about the project were provided in advance to the participants and they gave written informed consent before taking part. Ethics approval to conduct the experiments was in line with the ethical principles of the Helsinki declaration of 1975 and obtained from the Faculty of Science and Engineering Research Ethics Panel (FREP/DREP: 0218–03) at Anglia Ruskin University. Participants were reimbursed for time spent and compensated for travel expenses if required. Participants sat on a comfortable chair and placed their heads in a chin- and forehead rest. Before an experiment for a participant began, the stimuli were aligned to ensure comfortable viewing with both eyes. A recently established 4-AFC task^29^ was used to indicated whether an exclusively horizontal, an exclusively vertical, completely superimposed, or a piecemeal percept was seen and indicated through pressing and holding a buttons in accordance to those 4 options. In case of IOG paradigm, piecemeal entails both percepts in which portions of both stimuli were perceived but also in which just a split-grating of one eye was seen (‘eye-of-origin percept’), for readability and comparability with CBR we refer to those as piecemeal as well. The experimental trial was initiated by the participant pressing one of the buttons to starts the trial and then ongoingly reported perception via the appropriate button press option. The participants were instructed to observe the centers of the gratings. Prior the actual experiment, training trials were performed to familiarize the participant with the task.

One experimental session was carried out and included 8 trials per condition (48 trials in total): contrast 0.78 vs 0.78, 0.08 vs 0.08, and 0.50 vs 0.08. During the study, the split-grating’s location for each eye (IOG experiment) and grating orientation (IOG and CBR experiment) were counterbalanced for all conditions. Each trial lasted at least 60 s. Breaks between trials were permitted whenever desired and a longer break between the IOG and CBR experiment were given. Thus, a complete session lasted between 60 and 90 min, depending on the breaks for each participant. Participants recruited outside of CARD were asked to answer the long AQ as well as EQ questionnaires and asked whether they had been diagnosed with autism by a clinician.

### Data analysis

#### Raw data and pre-processing

Raw data were stored in .mat-files and Matlab (2021a) was used to write customized codes for the analysis and visualization of the data in the current study. We first structured the raw data by participant, condition including contrast and grating orientation, group and paradigm. Response durations ≤ 180 ms were excluded to avoid responses that are unlikely due to a reaction time limitations^56^ and we excluded the last response as trials were not stopped at 60 s but ended after a button was released (> 60 s).

#### Basic statistics

We analyzed data within each trial, across trials within a condition, and across grating orientations when calculating exclusive visibility and interocularly grouped percepts. Basic statistics were calculated for each perceptual state, namely relative proportions in percentage and mean perceptual durations in seconds. The total perceptual durations of exclusive events (i.e. sum of responses for a horizontal and the vertical percept), superimposition, and the other percepts across the individual trial duration and their respective mean durations were calculated. Also, perceptual alternations, termed flips, were calculated, including full and half flips. Full flips were defined as changes from one exclusive percept to another without any intermediate percept. All other possible perceptual changes were defined as half flips.

Comparison between autism and control groups were calculated using independent two-tailed t-tests, using Matlab2021a.

#### Analysis of perceptual phase distributions

The distributions of the exclusive phase durations were fitted with a gamma function. For each experimental condition and each subject, data was first normalized by dividing the phase durations by the relevant mean. These normalized data were then combined across subjects. The perceptual phases are presented in the following form using a gamma distribution:

$${{f}}\left( {{{x}}|\upalpha ,\upbeta } \right) = \frac{1}{{\upbeta ^{\alpha } \Gamma \left( \upalpha \right)}} {x}^{{\upalpha - 1}} e^{{\frac{{ - {{x}}}}{\upbeta }}} ;{{x}} > 0, \upalpha > 0, \upbeta \ge 0$$

where the “shape” parameter represents the skewness of the distribution, the “scale” parameter scales the distribution along the abscissa corresponding to the number of perceptual events. The coefficient of determination (R^2^) was used as a measure of goodness-of fit^57–59^. The X/Y locations were calculated to estimate the peak latencies and amplitudes of the Gamma functions. A trapezoidal numerical integration was used to estimate the areas under the Gamma functions for an x-axis length from 0.18 to 4 in 100 steps.

#### Linear function model to correlate AQ, EQ and multistability measures

We correlated AQ (EQ in supplementary materials) data for each group and paradigm separately, calculated linear fits using the Matlab function *fit*, thereby testing using one-sample t-tests whether the slopes of the functions were significantly different from baseline 0, and for mean phases durations, i.e. mean duration of all percepts and all alternations for all contrast conditions. Moreover, we report parameters that have been reported in the past to be predictive of autistic traits^31,60,61^ in CBR, namely mean exclusive durations, full and half flips, and additionally grouping, piecemeal, and superimposition mean durations (supplemental materials).

#### Mapping of weighted Hidden Markov transition probabilities

#### Weights

We first extracted the durations of each actual-to-posterior change until the second last response, and then calculated their means for each pre-processed trial using MatlabR2021a. For example, assuming a trial in which the first percept seen was ‘piecemeal’, the second ‘vertical exclusive’, we would calculate the average duration of ‘actual’ piecemeal state before flipping to ‘posterior’ vertical and assigned it to ‘piecemeal-to-vertical’. Note that the last response was excluded as it cannot have a posterior. Then, we averaged those actual-to-posterior states across trials, participants and conditions. For the final graph (Fig. 2), we excluded same-state alternations that were due to the extractions of brief durations (< 180 ms, see methods) from the raw data. The nodes in the graphs within Fig. 2 represent mean durations for each perceptual state, averaged across trials, participants, and conditions for each paradigm and group. The diameter of the nodes is related to the length of mean durations, i.e. the larger the diameter, the longer the respective mean duration. The line thickness of each transition is linked to the weight duration of the specific actual-to-posterior state and as for the node diameter indicates the length of that state before transitioning.

#### Transition probabilities

We calculated maximum likelihood estimates for each trial using MatlabR2021a’s Hidden Markov Model algorithm *hmmestimate*, then averaged those across trials, participants, and conditions. This step was followed by applying the discrete-time Hidden Markov chain algorithm *dtmc* and *graphplot* to visualize the transitions. We then tested whether the probabilities were normally distributed using the chi-square test and depicted those results within each Hidden-Markov-Chain-Graph (Fig. 2). Kullback–Leibler divergence tests (*KLDiv*) were used to compare transition probabilities for autism and controls for both paradigms. The closer its result is to zero, the more identical the compared distributions.

#### Comparison tables of weighted transition probabilities

**Figure 3:**
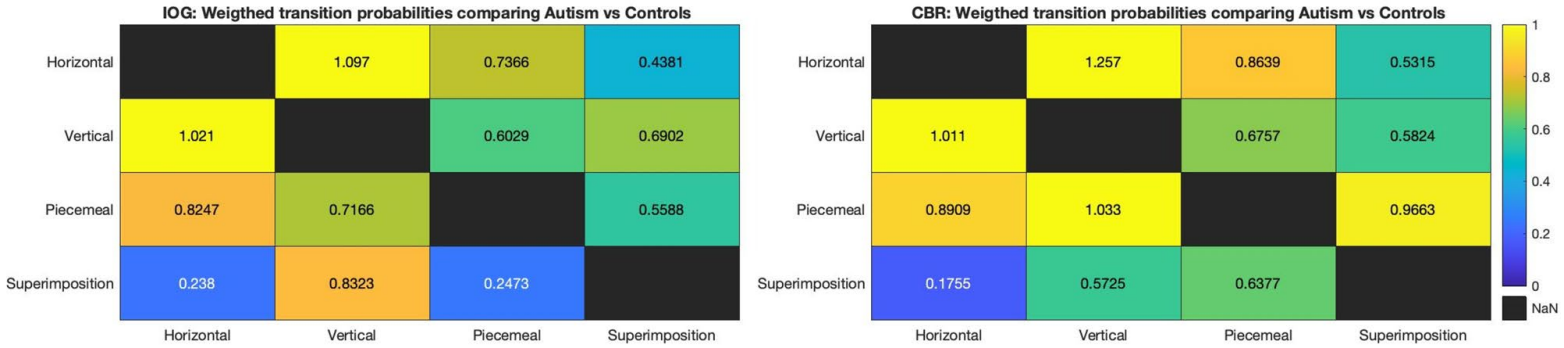
Comparison of weighed transition probabilities between autism and control group for IOG (left) and CBR (right). The colour bar indicates the degree of difference for each state, expressed as transitions from Y to X axis, since these transitions are asymmetric.

As a result of the weight and probability calculations, we had for each group and paradigm a 4 × 4 transition probability and an Actual-to-Posterior weights matrix. We divided each transition probability with its respective Actual-to-Posterior mean duration, resulting in weighted transition probability matrixes for each paradigm and group. In the next step, we divided each weighted transition probability for autism-IOG by control-IOG and autism-CBR by control-CBR and plotted those in two heatmap tables (Fig. 3). As these were unconventional data, not gained via a standard method, we used a non-parametric approach via bootstrapping to test the significance of difference between those tables. We generated means of 12 values, incrementally increasing from 0 to 1 and resampled those 10,000 times with replacement. We calculated the mean (0.5) and standard deviation (0.0863) of that simulated distribution and used a z-test to find out whether the difference between the two tables was significantly different from chance.

## Results

We first report the Hidden Markov chains, comparisons of weighed transition probabilities between groups and paradigms, but also report findings using traditional analysis methods. Next to exclusivity results generated while using the CBR paradigm, we report for the first time findings for piecemeal and superimposed percepts in autism. In the IOG paradigm, piecemeal defines both percepts in which portions of both stimuli were perceived but also in which a bipartite-grating of one eye was seen (‘eye-of-origin percept’), for readability and comparability with CBR we also refer to those percepts as ‘piecemeal’. Then, we correlated AQ and EQ (see supplementary materials) questionnaire findings with the results of the current study. At the end of the result section, we provide a breakdown of each contrast condition’s result.

### Transition probabilities and temporal dynamics of IOG and CBR perception

#### Within group and paradigm analysis using Markov Chains

Hidden Markov chains depicted in Fig. 2 represent the following information: maximum likelihood estimate of transition between each state using colour, all transitions, and their directions, indicated via arcs with arrows, their respective mean durations, expressed via line thickness, and the mean durations of each perceptual state, shown by the diameter of the nodes. For example, Fig. 2A depicts a summary across contrast conditions, autistic participants and trials for the IOG paradigm and shows that the node for piecemeal perceptual experiences has the largest diameter meaning that it had the longest mean duration (3.7 s) compared to horizontal (3.2 s), vertical (1.7 s) and superimposed (1.7 s) perception. The arc with an arrow from piecemeal to vertical node in the same graph indicates that such transition occurred, its width indicates a mean actual-to-posterior duration (4.4 s), and its turquoise color refers to the probability (0.48) of a change in this particular direction determined by the Hidden Markov chain algorithm. The chi-square p values inset on the left-hand side are variance test results and show whether the transition probability was significantly different from a random probability, in this case 0.33 (i.e. piecemeal would in 33% of the time change either to horizontal, vertical, or superimposition). As indicated by those chi-square values in Fig. 2, the transitions were not random. We also compared the probability distributions across contrast conditions for autistic and control group using the Kullback–Leibler divergence test for difference of distributions, applied to each of the four perceptual states. We found that CBR’s the difference between autistic and control group’s maximum likelihood transition distributions was largest for horizontal (0.021) states whereas the largest difference for the IOG paradigm was found for piecemeal (0.038).

#### Between groups comparison of weighted IOG and CBR transition probabilities

We used the transition probabilities and weights for each condition to compare the two paradigms with each other as shown in Fig. 3. Specifically, we divided the transition probabilities by the weights for each cell to gain weighted probabilities and then compared autism with control for each paradigm by dividing the autism group’s weighted probabilities with those of the control group for both paradigms. We compared the two tables by applying a bootstrapping technique of means (10,000 samples between 0 and 1) and tested whether the differences between IOG and CBR in Fig. 3, falls within 95% of the bootstrapped distribution. A z-test revealed that the differences between those tables were not within 95% of the normal distribution created via bootstrapping, in other words, the difference is unlikely to be a random result but rather a true difference [p < 0.001].

#### Relative proportions of perceptual states

**Figure 4:**
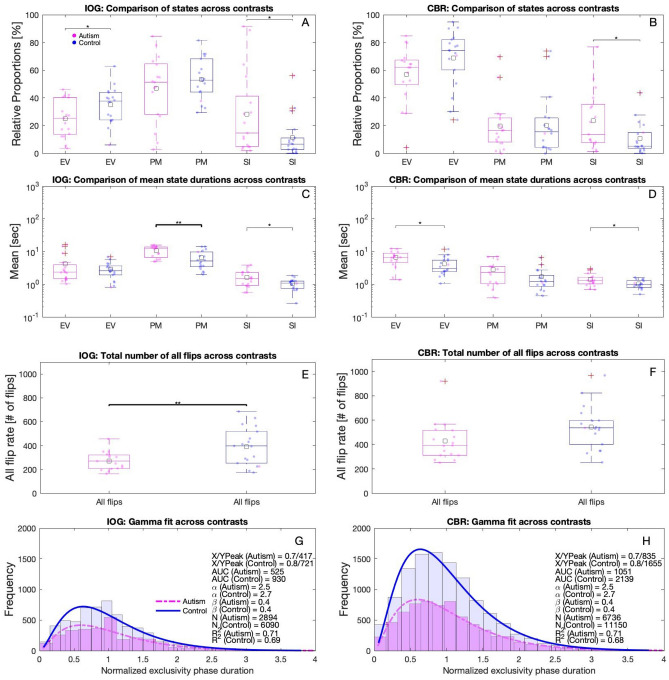
Summary of traditional measures for IOG (left) and CBR (right), for both autism (magenta) and control (blue) group. Averages across trials, participants, and conditions for relative proportions (**A**, **B**), mean durations (**C**, **D**), sum of all perceptual flips (**E**, **F**), and Gamma function fits to normalized exclusive IOG and CBR percepts with their respective histograms (**G**, **H**) are depicted. ‘EV’, ‘PM’, and ‘SI’ in A-F refer to sum of left and right exclusivity, piecemeal, superimposition, respectively. The scattered dots indicate data for each individual, squares depict the means, boxes the interquartile ranges (25th-75th percentiles), horizontal lines within each box the medians, whiskers extend to the extreme values, outliers are plotted outside the whiskers. Using 2-tailed independent t-tests, ** and * refer to statistically significant difference of* p* < 0.01 and* p* < 0.05, respectively. On the right-hand side of G and H, the following parameters for each group are depicted: shape *a* and scale *b* parameters of the Gamma functions, numbers of events N, coefficients of determination R ^2^, areas under the Gamma function curves (AUC) from 0.18 to 4 along x axis, X/Y peaks of Gamma functions.

Proportions of IOG-exclusivity was significantly greater for the control than for autism group t(33) = − 2.2, p < 0.05 and the same trend, although not significant (*p* = 0.096), was found for CBR. Significantly greater proportions of superimposition were found for both IOG t(33) = 2.1, *p* < 0.05 and CBR t(33) = 2.2, *p* < 0.05 paradigm. Piecemeal states were not significantly different for both IOG and CBR (*p* > 0.05). CBR generated more exclusivity compared to IOG (CBR 57% ± 21 standard deviation and 69% ± 20 (autism and control group, respectively) vs. IOG 25% ± 14 and 35% ± 13) (Fig. 4A–B). The reversed picture appears for piecemeal with high piecemeal proportions for IOG (47% ± 24 and 54% ± 15) but not for CBR (20% ± 18 and 20% ± 21).

#### Mean durations

Mean durations tend to be longer for the autism participants compared to controls for both paradigms (Fig. 4 C-D). Specifically, autistic participants mean exclusivity durations using the CBR paradigm were longer compared to those of controls (6.7 s ± 3.3 vs. 4.2 s ± 2.7 t(32) = 2.5, p < 0.05) and the same trend was found for IOG (4.3 s ± 4.8 vs. 2.9 s ± 1.6), however it reached no statical significance t(32) = 1.1, p > 0.05. Piecemeal percepts showed a similar pattern: IOG 10.7 s ± 4.0 vs. 6.4 s ± 4.0, t(32) = 3.1, p < 0.01 and the same trend but not significant for CBR 2.8 s ± 2.2 vs. 1.7 s ± 1.6, t(30) = 1.7, p > 0.05. Similarly, phases of superimposition were significantly longer for the autism group for both IOG (1.6 s ± 1.0 vs. 1.1 s ± 0.4; t(33) = 2.4, p > 0.05) and CBR paradigm (1.4 s ± 0.7 vs. 1.0 s ± 0.3; t(32) = 2.2, p > 0.05).

#### Flip rates

We analyzed differences of perceptual alternations between autism and controls as seen in Fig. 4E–F. We summed all flips across conditions within each group and experimental paradigm, resulting in significantly more alternations for the control group compared to autism using and IOG t(33) = − 2.8, p < 0.01. CBR showed the same trend however not reaching statistical significance t(33) = -2.0, *p* = 0.055 experimental paradigms.

#### Distributions of exclusive phases

IOG produced fewer exclusive events for each group than CBR did (Fig. 4 G-H). The distributions for IOG and exclusive CBR percepts are well fit with a gamma function as indicated by the R^2^ values. We also calculated the lognormal functions for IOG and exclusive CBR percepts as it has been reported that it fits as well and sometimes better to CBR data^62^, but none of those fit as well as the Gamma function, with R^2^ values for IOG of 0.71, 0.69 and CBR of 0.71, and 0.68 for autism and control group, respectively. The estimates of area under the Gamma function curves were larger for the controls compared to the autistic group for both paradigms.

The Gamma functions for autism had a lower latency and overall lower amplitude in both paradigms compared to controls as indicated by X/Y peaks in Fig. 4G–H. These trends were the same for all three contrast conditions and driven by the higher number of percepts during the same contrast conditions (see appendix).

#### Correlation between IOG parameters and autistic symptomology measure AQ

**Figure 5:**
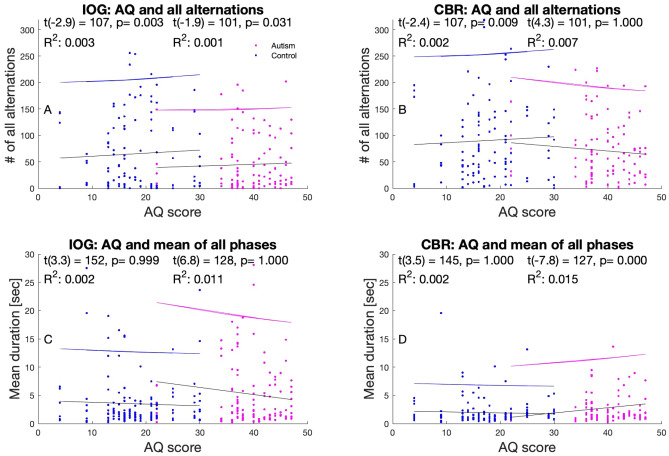
Scatterplots for IOG (left) and CBR (right), for control (blue) and autism (magenta) participants, for all alternations (**A**, **B**) and all mean phase (**C**, **D**) durations are depicted including all contrast conditions.. Included are linear fits (solid magenta and blue lines), coefficient of determination R^2^and 95% upper confidence intervals (dotted lines). The statistics of one-sample t-tests were included too, indicating the difference to a random sample.

We tested how well AQ values correlated with IOG and CBR measures (Fig. 5A–D), namely all mean phase durations and all alternations (see detailed breakdown for full flips, half flips and exclusive, piecemeal, and superimposed mean phase durations correlated with for AQ and EQ in supplementary materials).

To do so, we calculated linear fits, 95% confidence intervals, and goodness-of-fit as expressed with R^2^ and tested whether the slopes of the functions were significantly different from 0. We found significant correlations for both groups and number of alternations during the IOG experiment, the same result was found for only the control using CBR. The mean phase durations increased significantly with increasing AQ for the control group only using the CBR paradigm (Fig. 5D).

#### Comparison of contrast conditions

Here, we report detailed breakdown of the data shown in the Fig. 4, including summary statistics for each contrast condition of mean phase duration, relative proportions, and perceptual alternation counts, for namely full as well as half flips (Table 2).

**Table 2:** Summary of mean phase durations and relative proportions of exclusive, piecemeal, and superimposed perceptual states averaged across trials and participants, and the sum across trials and participants of full and half flips for each contrast condition and both paradigms comparing the results for the autism and control group.

	Mean phase ± σ [sec] % proportion ± σ Exclusivity Autism vs. Control	Mean phase ± σ [sec] % proportion ± σ Piecemeal Autism vs. Control	Mean phase ± σ [sec] % proportion ± σ Superimposition Autism vs. Control	Full flips [sum] Half flips[sum] Autism vs. Control
IOG Low contrast	[sec]3.0 ± 3.0 vs. 1.7 ± 0.7 [%]18.3 ± 13.5 vs. 24.6 ± 11.0	11.3 ± 5.0 vs. 8.3 ± 7.8 57.1 ± 32.5 vs. 63.4 ± 22.4	1.4 ± 1.2 vs. 1.1 ± 0.4 s 24.6 ± 37.2 vs. 12.0 ± 20.3	82 vs. 154 1072 vs. 2252
CBR Low contrast	6.6 ± 4.2 vs. 4.6 ± 4.4 67.8 ± 14.8 vs. 74.2 ± 12.7	3.6 ± 4.6 vs. 1.5 ± 1.4 19.5 ± 21.4 vs. 17.3 ± 21.1	1.4 ± 0.5 vs. 1.1 ± 0.4 12.7 ± 16.8 vs. 8.5 ± 15.1	489 vs. 864 1424 vs. 2213
IOG High contrast	2.0 ± 2.4 vs. 1.3 ± 0.6 18.1 ± 9.6 vs. 20.0 ± 7.3	12.6 ± 7.4 vs. 7.4 ± 5.1 58.0 ± 27.8 vs. 69.4 ± 21.5	1.9 ± 1.9 vs. 1.2 ± 0.6 24.0 ± 31.5 vs. 10.6 ± 21.0	96 vs. 190 1116 vs. 2240
CBR High contrast	3.3 ± 3.3 vs. 2.2 ± 1.2 58.7 ± 16.1 vs. 65.6 ± 13.3	3.0 ± 3.3 vs. 2.0 ± 2.1 28.4 ± 23.7 vs. 29.6 ± 25.6	1.8 ± 1.2 vs. 1.0 ± 0.5 13.0 ± 24.8 vs. 4.8 ± 5.8	958 vs. 1108 2143 vs. 3350
IOG Low vs. High contrast	Low: 1.2 ± 11.8 vs. 0.8 ± 6.4 High: 9.8 ± 8.4 vs. 7.9 ± 5.8 Low: 3.0 ± 6.7 vs. 1.9 ± 4.4 High: 36.9 ± 29.3 vs. 58.0 ± 23.1	5.8 ± 9.0 vs. 3.2 ± 2.4 22.6 ± 21.8 vs. 29.1 ± 23.6	1.6 ± 1.4 vs. 1.0 ± 0.4 37.5 ± 37.5 vs. 11.0 ± 18.9	209 vs. 183 2016 vs. 1999
CBR Low vs. High contrast	Low: 1.5 ± 15.7 vs. 1.5 ± 5.6 High: 18.7 ± 15.1 vs. 12.9 ± 7.3 Low: 3.9 ± 6.8 vs. 6.9 ± 4.7 High: 41.7 ± 27.8 vs. 65.0 ± 19.6	2.7 ± 3.2 vs. 1.9 ± 1.6 11.0 ± 17.2 vs. 12.6 ± 17.5	1.3 ± 0.7 vs. 0.9 ± 0.3 43.5 ± 38.7 vs. 15.5 ± 17.7	413 vs. 279 1842 vs. 1848

As shown in the top portion of each dedicated cell in Table 2, people with autism experience longer phase durations for all contrast conditions and for both paradigms compared to the control group. The results for relative proportions are variable across contrast conditions (Table 2, lower portion of each dedicated cell), however it is noteworthy that the higher overall degree of superimposition’s relative proportions and mean phases durations shown in Fig. 4 was true for all contrast conditions, but with a large variability. People with autism experience fewer alternations when the contrast is bilaterally the same, but may increase above that of controls when the contrast is varied unilaterally. The CBR generated for each contrast condition more perceptual alternations than the IOG paradigm.

## Discussion

### Markov chains as a tool to analyze and visualize multistable perception

Markov processes are defined by changes of discrete events across time that are dependent upon the current state. Markovian models have been used in previous computational studies to simulate data that resemble that of perception during CBR^63,64^, IOG^48^ and other multistable figures^65^. One study used a Variable-Length-Markov-Chain technique to test for discrepancy from randomness in various multistable figures^66^, however, to the best of our knowledge, no study has applied Markov chain models to analyze and visualize transition probabilities between perceptual states for CBR or IOG data, nor accounted weights for each transition, nor used this approach to investigate differences between neurotypicals and autistic individuals. The Markov chain model used in the current study (Fig. 2) has several advantages over conventional approaches (Fig. 4) for both within-group and between-group analyses. The visualization of the chains allows the comprehensive depiction and statistical comparison of within-group and paradigm results, i.e. which transition occurred, the mean durations of each state, the mean durations prior their alternation to another state, and how likely each transition was. The model estimates the maximum likelihood of perceptual transitions, and a chi-square test showed that the distributions were non-random. In a second step, we calculated weighted transition probabilities by taking into account each percept’s mean duration before changing to the next state, here referred to as actual-to-posterior weights. We divided each of those autism group weighted transitions by those of the control group and depicted them in tables in Fig. 3. In this way, we compared the results between paradigms using a bootstrapping statistical approach to investigate whether the results gained via CBR and IOG paradigms are different and found that their difference was not within 95% of a normal distribution, thus implying that it is indeed an actual difference between those paradigms. When comparing the within each paradigm the two groups’ probabilities using Kullback-Leibner divergence tests, we found that the largest differences between states, differed between paradigms as well, further distinguishing the two paradigms from another.

Markovian transition probabilities are memory-free (unlike Bayesian approaches), and as other researchers suggested, binocular rivalry may be fall into the same category e.g.^67,68^ , hence, it may offer an alternative computational way to predict behavior of states alternating across time, such as those during multistable perception. Indeed, previous computational work was able to replicate behavioral findings during binocular rivalry using Monte Carlo simulations based on Markovian principals^63^. On the other hand, Bayesian ideas have been proposed to explain alternation of other multistable percepts^69^ and relatedly, statistical work, which shows that the cumulative history of perceptual states is predictive of future events^70^ challenge the view of a stochastic process. We show that for both groups and paradigms all possible transitions occurred and that the transition probabilities between groups and paradigms were similar (Fig. 2), but that their weights for each transition as expressed in mean durations were significantly different (Fig. 2 nodes and arrows thickness differences; Fig. 3). Specifically, greater differences in weights for both paradigms (i.e. arrow and nod thickness in Fig. 2) were notable in autism compared to controls, reflecting potential differences in inhibition and excitation.

#### Piecemeal and superimposition in autism

Visual functions have been studied intensively in autism^1,4,5^, including CBR^30–32,34,35^. Our study had a comparable sample size in relation to previous CBR studies and, as reported above, the CBR findings of mean durations, and perceptual alternations and correlations are in line with those studies, with the exception of one study^30^ that found only a non-significant trend towards more mixed percepts for autism, a result that is potentially explained by the choice of a small stimulus size of 1° as it known that small grating stimuli bias results toward exclusivity^20^.

Sensory differences in autism, including visual perception, have been thought to be a consequence of an excitation/inhibition imbalance across the cortex^15–17^ and as a consequence have been suggested to cause longer durations of mixed perception in previous CBR studies^31,32^. To the best of our knowledge, this is the first study that measured both piecemeal and superimposition percepts in autism. When averaged across trials, participants, and contrast conditions, we autistic participants perceived significantly longer mean phase durations of piecemeal and superimposed percepts using the IOG paradigm and exclusive and superimposed percepts using the CBR paradigm (Fig. 4C–D). All mean durations for each single contrast conditions generally tended to be longer in autism compared to the controls (Table 2). Consequently, people with autism experienced fewer perceptual alternations in both paradigms (Fig. 4E,F). The relative proportions of exclusivity were significantly longer for IOG and trended toward the same direction in IOG and notably superimposition was significantly elevated in both paradigms for the autism group (Figure A-B).

Robertson, Ratai and Kanwisher ^33^ found that glutamate levels (an excitatory neurotransmitter) in early visual cortex correlated strongly with perceptual suppression for both autism and control groups during a CBR task, while GABA levels (an inhibitory neurotransmitter) were only correlated in controls, resulting in fewer proportions of dominant percepts and fewer alternations for the autism group. Our results agree with findings by Robertson et al. who showed that exclusive percepts were not as often formed for autistic individuals as for controls (Fig. 4G–F), but once formed, exclusive percepts tended to remain for a longer period of time (Fig. 4B–C). In the light of the findings of Robertson et al. ^33^, we suggest that difference was due to lacking inhibitory counterforce. This also explains the longer mean phases of piecemeal perception in autism. It is noteworthy that we used the term piecemeal percepts during IOG paradigm to have a comparison to the CBR task, but during IOG, piecemeal could be either piecemeal or eye-of-origin percepts. We do not have data that further disentangle those states but given that both types have been suggested to be fully (eye-of-origin^71^) and partially (piecemeal, i.e. local zones of rivalry^20^) a process within early visual cortex, it may suggest a greater role of early visual cortex when using an IOG paradigm. Superimposition has been thought of as a perceptual marker for binocular fusion^21,23^ and atypical binocular vision in autism has been reported previously^2,37–42^, We did an optometric screening prior the experiment to exclude such confounding factors. Counter to our hypothesis, we found that superimposed perception occurred longer and to greater proportions in autism than in controls (Fig. 4A–D, see also Table 2). One potential explanation may be that the lack of inhibition but regular excitation in autism as reported by Robertson and colleagues^33^ extends to the binocular processing sites, i.e. once the state is active it remains longer. An alternative explanation may be that the experienced superimposition was indeed superimposition without fusion, known in other clinical contexts as double vision or diplopia, thereby giving rise to both single percepts while not suppressing either of them.

#### Autism traits correlated with IOG and CBR measures

Previous studies showed that CBR measures such as alternation rate or mixed mean durations correlated well with the symptomology of people with autism when using foveally presented stimuli^31,60,61^. We found that overall alternation rates increase with increasing AQ scores for all except the autism group when using the CBR paradigm (Fig. 5A–B). CBR’s mean phase durations increase with increasing AQ score for autism otherwise these trends did not differ significantly from zero (Fig 5C-D). The interested reader will find a detailed breakdown for different flip types and separate mean durations for AQ and EQ in the supplementary materials.

#### IOG and weak-central-coherence theory of autism

According to both the Weak-Central-Coherence theory^11^ and the hyper-systemizing theory^72^, autistic people have a bias in processing local rather than global information. Our results agree with these theories as fewer IOG percepts were formed by people with autism compared to controls (Fig. 4), which in turn is linked to the E/I imbalance theory of autism as explained above.

In addition to the explanation based on reduced GABA levels, we suggest a second potential reason. Opponency neurons, a class of neurons that receives excitatory input from one eye and inhibitory input from the other eye for each orientation^46^, have been suggested to be the correlate that forms the coherence, i.e. exclusive IOG perception^47^. Opponency neurons are therefore a different substrate from mutually inhibiting monocular neurons^73^ that are the likely mediator of eye-of-origin percepts (part of IOG piecemeal), for exclusive CBR percepts and locally rivalry (parts of IOG piecemeal and all CBR piecemeal). We speculate that similar as for interneurons^74^, opponency neurons may be disrupted in autism causing fewer exclusive IOG events (Fig. 4G–H) compared to the neuro-typical group.

#### CBR and IOG paradigms compared

Autistic people showed fewer perceptual flips during grating-induced CBR in previous studies^31,32^ and also when using other multistable paradigms^75^ compared to neurotypical people. We replicated those findings for CBR and extended them to IOG paradigm (Fig. 4F,E, respectively). Moreover, there were key differences between perceptual experiences elicited by CBR and IOG, even though the same stimulus contrasts were presented to each eye with both paradigms. There were fewer exclusive percepts (Fig. 4G,H) and perceptual flips (Fig. 4E,F) for IOG than for CBR for both autistic and neurotypical participants. This observation is in agreement with previous studies using colored dot stimuli in a neurotypical cohort^44^. The distribution of exclusive percepts for both CBR and IOG paradigms were fitted well with the Gamma function in previous studies^44,51^ in a general population and fitted also well for both groups the current study (Fig. 4G,H). As mentioned above, the analysis of the HMM also revealed a general difference between the two paradigms. It is possible that exclusive grating percepts in CBR and IOG could be created by a single late-stage pattern integrating mechanism that is invariant of eye of origin, however, this could not account for the differences in the dynamics of perception that are revealed by our various analysis techniques presented in this paper. Taken together, the formation of exclusive grating percepts during CBR is likely to underlie a different neural mechanism than that of exclusive percepts during IOG.

CBR has been suggested as a potential tool to aid in the diagnosis of autism^35^. We found more statistically significant differences between groups using the IOG paradigm compared to the CBR paradigm (Fig. 4). IOG quantifies next to complete (eye-of-origin; not directly investigated in this study) and partial (piecemeal) monocular suppression, binocular fusion (superimposition) and interocular grouping, each of which may involve potential distinct processing substrates. Further research is needed to disentangle the underlying processes of IOG percepts and to evaluate the paradigm’s validity as a diagnostic tool for autism research and therapy.

#### Other considerations

Levelt’s laws i.e., lawful interaction between physical stimulus changes and changes in perceptual dynamic, have been used to describe initially CBR^51,52^ and have been extended to IOG^47,48^. When considering the overall trends, the results show that increasing the contrast unilaterally increases the predominance (1st law) and the mean exclusivity duration (2nd law) in both groups and paradigms (Table 2). Interestingly the unilateral increase of contrast did increase the number of full flips for autism using the IOG paradigm, which is counter to the 3rd law whereas. Increasing the contrast bilaterally increased the alternation rate from one exclusive to another percept, here called full flip, in both groups and for both paradigms as well in line with the 4th law.

In the current study, the autism group (mean age: 39 years) was significantly older than the control group (29 years) (33) = 3.1, *p* < 0.01 using an independent two-sample t-test. A study between young (22 years) and elderly adults (60 years) and CBR^76^ showed that exclusive perception was significantly higher in the elderly group. It remains unclear whether this age difference in the current study was sufficient to contribute to the elevated mean exclusivity duration in the autism group as the autism cohort is substantially younger than the elderly group in the cited study.

#### Conclusion

The current study examined the dynamics of conventional binocular rivalry (CBR) and interocular grouping (IOG) in neurotypical and autistic adults. The results demonstrated that autistic people experience fewer exclusive IOG percepts, fewer perceptual alternations and longer mean durations compared to neurotypical controls. Relative proportions and mean phase durations during superimposed perception were significantly increased in autism for both paradigms. The maximum transition likelihoods as determined by a Hidden Markov Model demonstrated significant differences in the dynamic transitions among perceptual states between groups and further analysis revealed that transition distributions using the IOG paradigm were significantly different to those generated via a CBR paradigm. These results suggest that perception during IOG and CBR are processed by different neural mechanism. Markov chains are a useful tool to analyze multistable perception and have the potential to give new insights in the computational investigations of multistability.

## Supplementary Information

Supplementary Information 1.

Supplementary Information 2.

Supplementary Information 3.

Supplementary Information 4.

Supplementary Information 5.
