# Supplementary figures and images for: Visual consciousness dynamics in adults with and without autism
Source: Sci Rep. 2022 Mar 14;12:4376. doi: 10.1038/s41598-022-08108-0 (PMC8921201; doi:10.1038/s41598-022-08108-0)

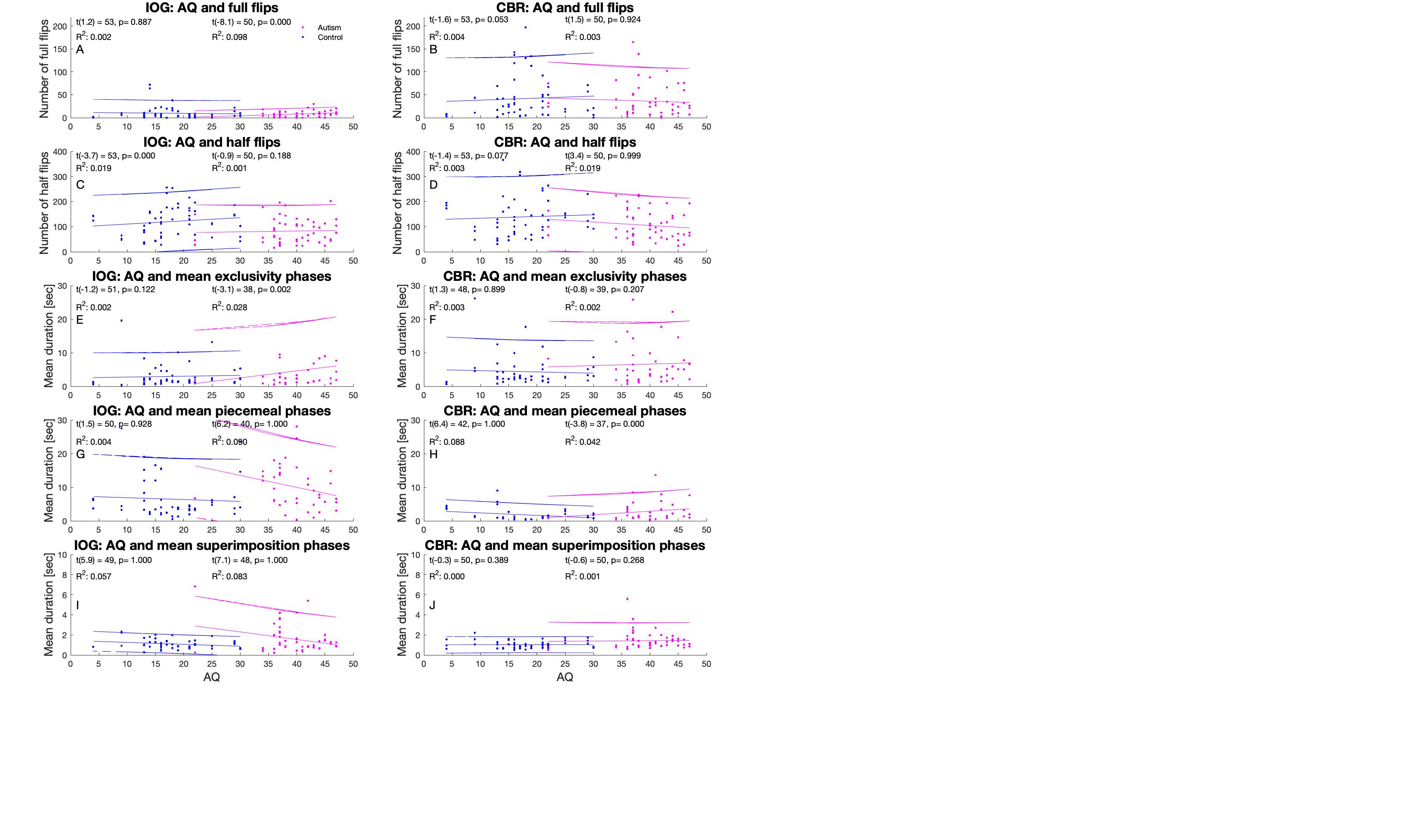

Supplement: Supplementary file 1 — Supplementary Information 1. [file 41598_2022_8108_MOESM1_ESM.jpg]

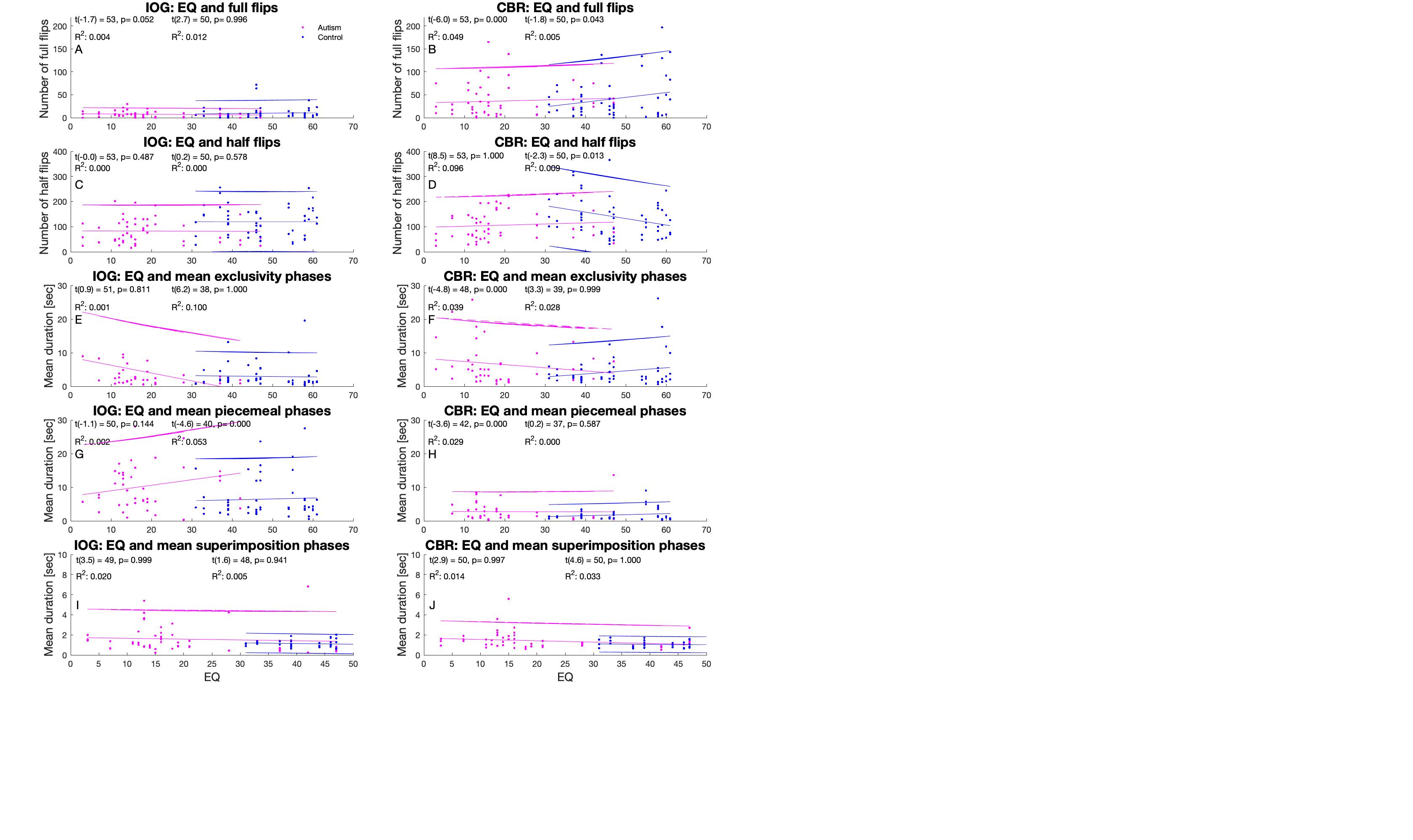

Supplement: Supplementary file 2 — Supplementary Information 2. [file 41598_2022_8108_MOESM2_ESM.jpg]

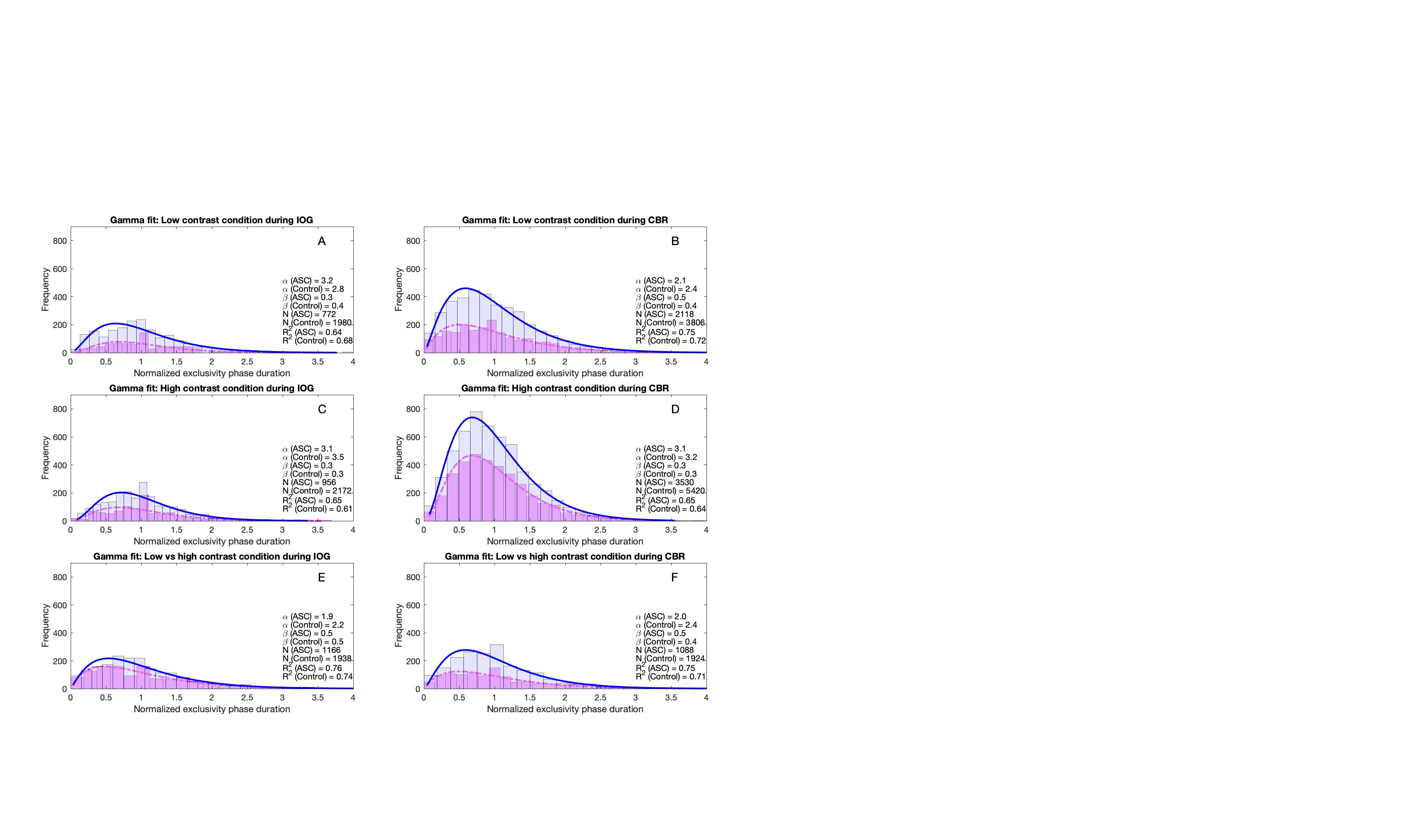

Supplement: Supplementary file 3 — Supplementary Information 3. [file 41598_2022_8108_MOESM3_ESM.jpg]

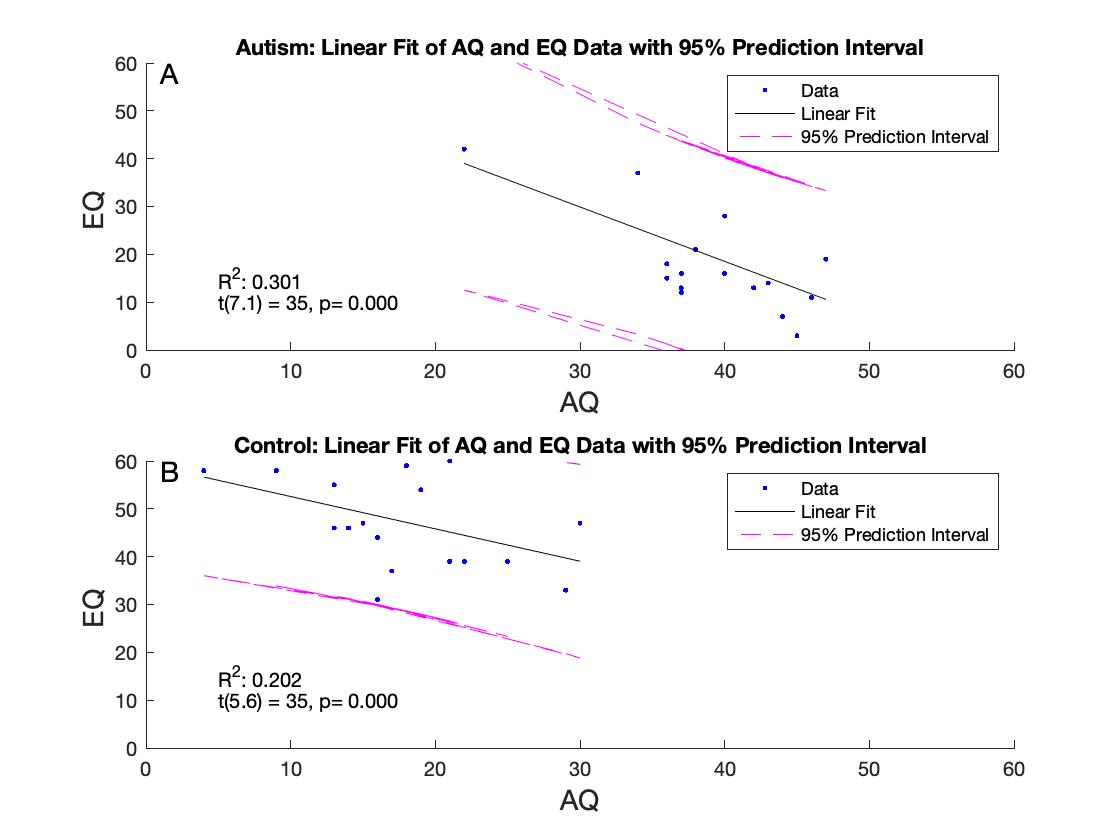

Supplement: Supplementary file 4 — Supplementary Information 4. [file 41598_2022_8108_MOESM4_ESM.jpg]
