# Supplementary material for: Visual consciousness dynamics in adults with and without autism
Source: Sci Rep. 2022 Mar 14;12:4376. doi: 10.1038/s41598-022-08108-0 (PMC8921201; doi:10.1038/s41598-022-08108-0)
Supplement: Supplementary file 5 — Supplementary Information 5. [file 41598_2022_8108_MOESM5_ESM.docx]

**Supplementary materials: Visual consciousness dynamics in adults with and without autism**

Jan Skerswetat* ^1,2^, Peter J. Bex^2^, Simon Baron-Cohen^3^

^1^ Anglia Vision Research, Department of Vision and Hearing Sciences, Anglia Ruskin University, Cambridge, UK

^2^ Department of Psychology, Northeastern University, Boston, USA

^3^ Autism Research Centre, Department of Psychiatry, University of Cambridge, Cambridge, UK

Corresponding Author *

Email address: j.skerswetat@northeastern.edu


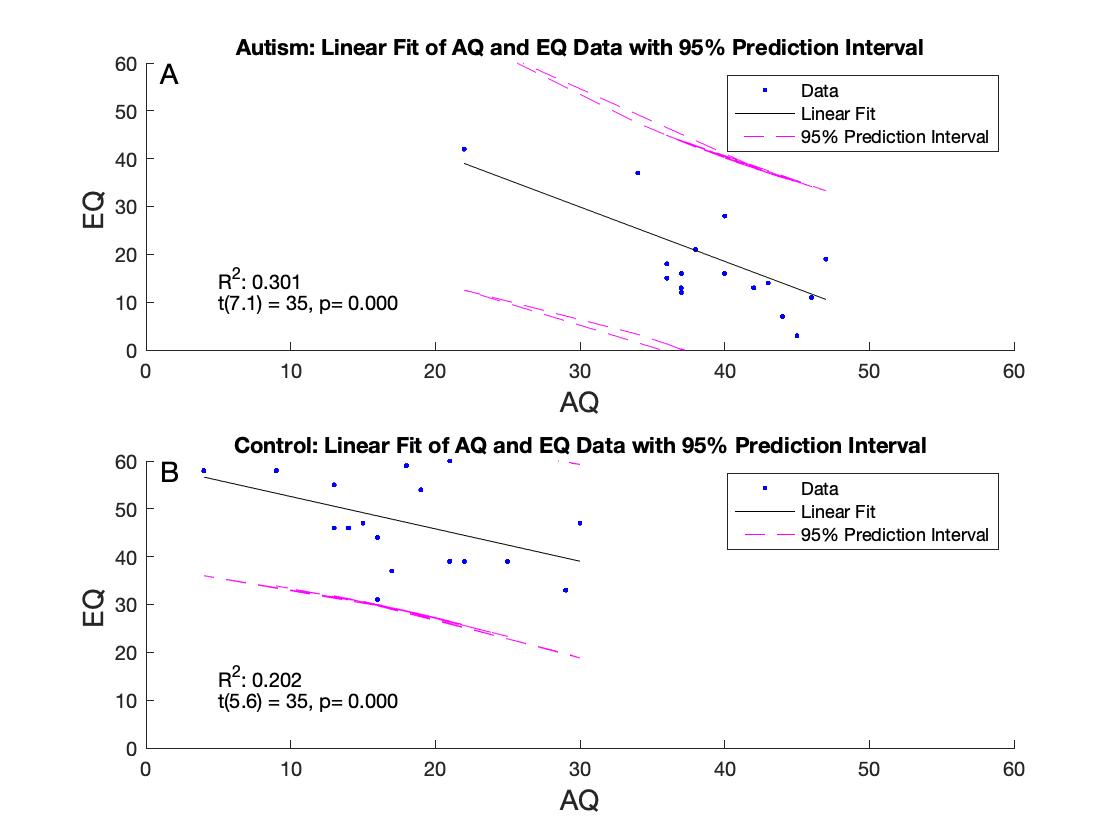


Figure 1: AQ (A) and EQ (B) correlation plot, including linear fits, coefficient of determination R^2^and 95% confidence intervals.


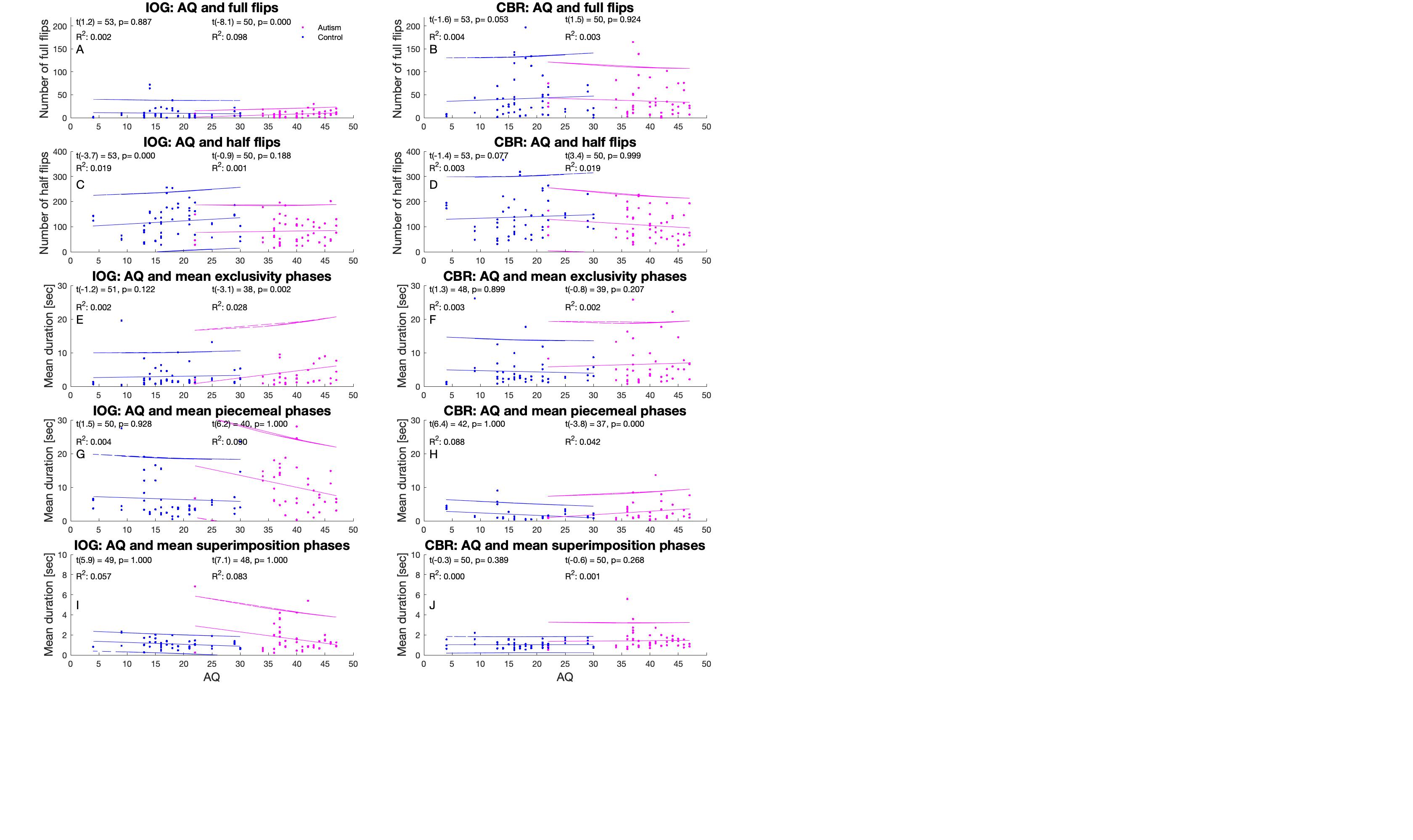


Figure 2: Scatterplots for AQ and IOG (left) and CBR (right), for Neurotypical and participants with autism, for full flips (A-B), half flips(C-D) and mean durations for exclusive horizontal and vertical percepts (E-F), piecemeal (G-H), and superimposition (I-J) are depicted including all conditions. Magenta circles represent the autism group, blue crosses represent the control group. Included are linear fits (black lines), coefficient of determination R^2^and 95% confidence intervals (black dotted lines). The statistics of one-sample t-tests were included too, indicating the difference to a random sample.

*
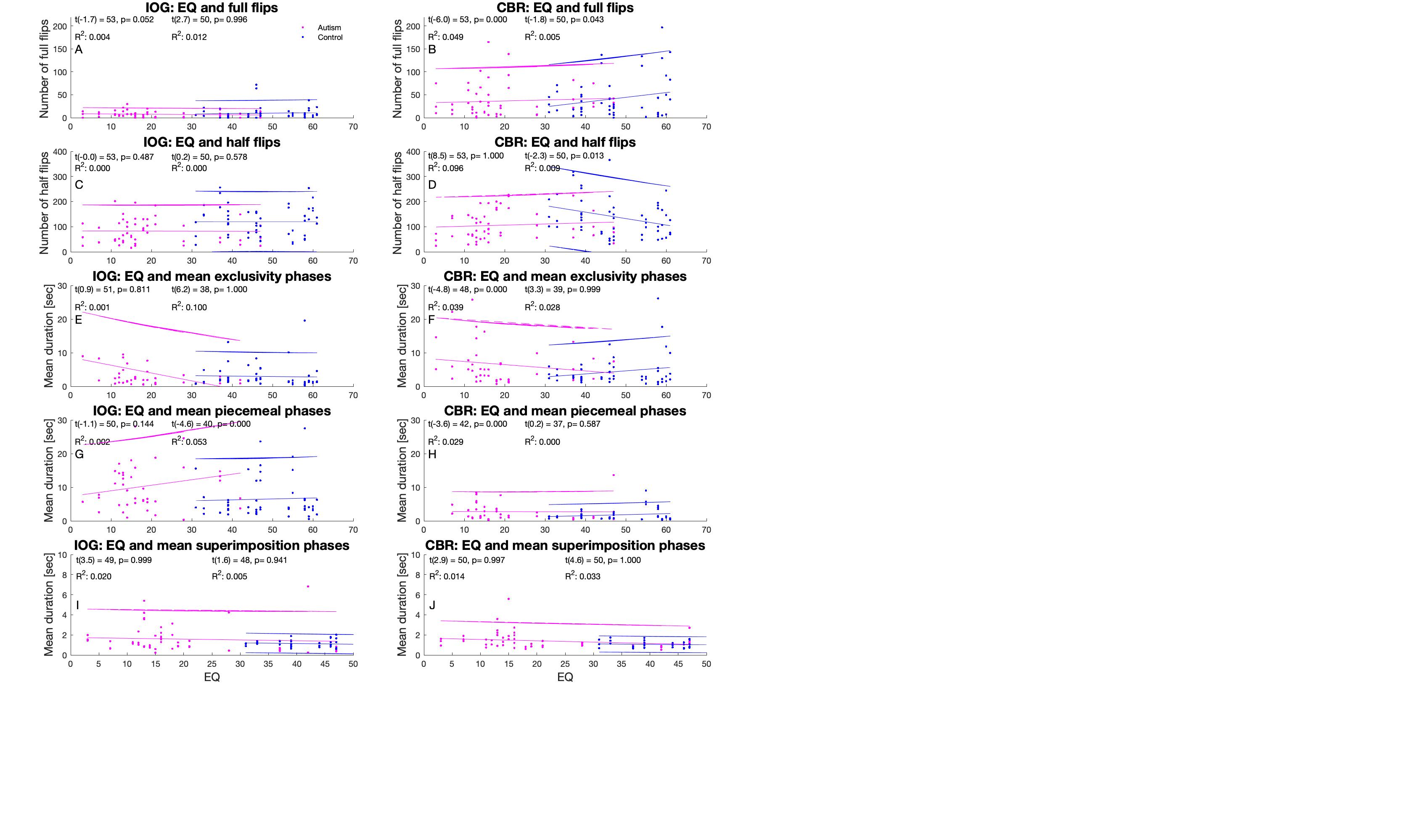
*

Figure 3: Scatterplots for EQ and IOG (left) and CBR (right), for Neurotypical and participants with autism, for full flips (A-B), half flips(C-D) and mean durations for exclusive horizontal and vertical percepts (E-F), piecemeal (G-H), and superimposition (I-J) are depicted including all conditions. Magenta circles represent the autism group, blue crosses represent the control group. Included are linear fits (black lines), coefficient of determination R^2^and 95% confidence intervals (black dotted lines). The statistics of one-sample t-tests were included too, indicating the difference to a random sample.

Figure 4: ﻿ Stimuli used to initiate interocular grouping (A-C) and conventional binocular rivalry (E-F). Orthogonally orientated high-contrast (A and D) and low-contrast (B and E) luminance defined gratings. High-contrast versus low-contrast condition is depicted in C and F. The contrast levels are indicated above each grating. ﻿The reader may experience both rivalry and grouping. Start with looking at the gratings in Fig. 1A reading distance. Position a fingertip halfway between the eyes and gratings, so that each eye views the fingertip centrally for one of the two gratings. Now fixate the fingertip with both eyes open. In the central patch of the now perceived three, overlapping gratings will compete and group perceptually.


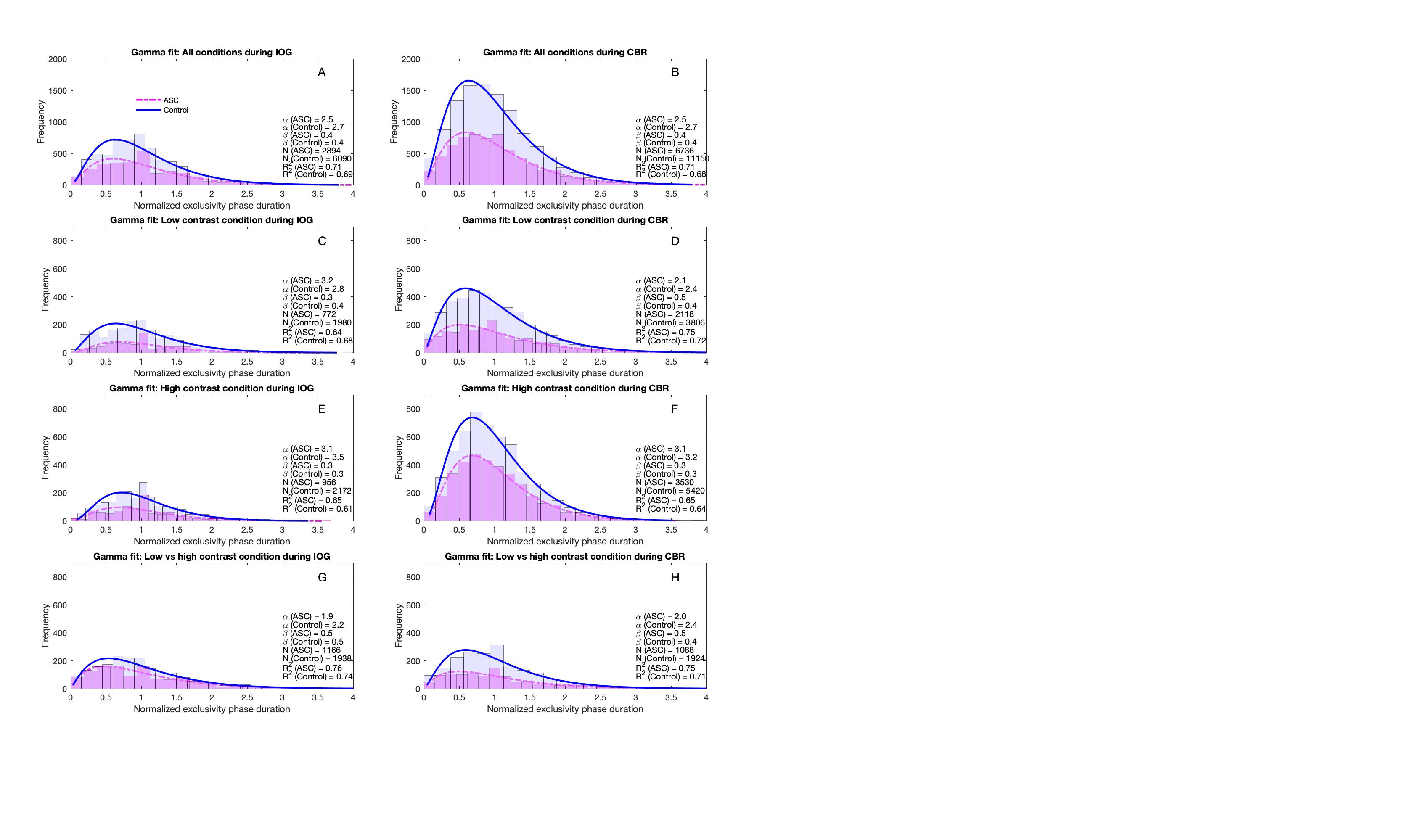


Figure 5: ﻿ Gamma function fits to normalized exclusive IOG and CBR percepts with their respective histograms for all contrasts combined (A-B), low vs. low (C-D), high vs. high (E-F), and low vs. high contrast condition are depicted.
